# PDL1 blockage increases fetal resorption and Tfr cells but does not affect Tfh/Tfr ratio and B-cell maturation during allogeneic pregnancy

**DOI:** 10.1038/s41419-020-2313-7

**Published:** 2020-02-12

**Authors:** Weihong Zeng, Shi Qin, Renjie Wang, Yuchen Zhang, Xiaoling Ma, Fuju Tian, Xiao-Rui Liu, Xiaoli Qin, Shujie Liao, Liqun Sun, Yi Lin

**Affiliations:** 1 Shanghai Key Laboratory of Embryo Original Diseases, The International Peace Maternity & Child Health Hospital, Shanghai Jiao Tong University School of Medicine, Shanghai, 200030 P. R. China; 2 Tongji Hospital, Tongji Medical College, Huazhong University of Science and Technology, Wuhan, 430030 Hubei P. R. China

**Keywords:** CD4-positive T cells, CD4-positive T cells, CD4-positive T cells, CD4-positive T cells, Reproductive disorders

## Abstract

A successful pregnancy requires sophisticated regulation of uterine microenvironment to guarantee the existence of semi-allogeneic conceptus without immune rejection. T follicular regulatory (Tfr) cells exert a suppressive effect on Tfh-cell expansion, B-cell response, and antibody production. Although accumulating evidence has demonstrated that dysregulations of Tfr cells can bring on various immunological diseases, their immunomodulatory roles during pregnancy still remain unheeded. Herein, we introduced an allogeneic normal-pregnant mouse model and found that CD4^+^CXCR5^hi^PD-1^hi^Foxp3^+^ Tfr cells were preferentially accumulated in the uterus at mid-gestation and displayed a distinct phenotype. In addition, the absence of PDL1 resulted in increased fetal resorption by favoring Tfr cells accumulation and upregulating PD-1 expression on these cells. However, PDL1 blockade affected neither the ratio of Tfh/Tfr cells nor the maturation and differentiation of B cells. Overall, our results are the first to present a correlation of Tfr cells accumulation with healthy allogeneic pregnancy and PDL1 blockade-induced miscarriage, and to indicate that appropriate assembly of Tfr cells is important for pregnancy maintenance. Since blockade of PD-1-PDL1 pathway leads to more Tfr cells and fetal losses, the reproductive safety must be taken into consideration when PD-1/PD-L1 checkpoint blockade immunotherapy is used in pregnancy.

## Introduction

A successful pregnancy requires extremely sophisticated regulation of uterine microenvironment to guarantee the existence of semi-allogeneic conceptus without immune rejection. Orchestrated balance of immune cells residing at the maternal–fetal interface fundamentally contributes to the immune defense protecting the host from external threats, as well as the maintenance of immune tolerance keeping the fetal from maternal immune attack^1,2^. An in-depth exploration into the unique immunological alterations during pregnancy will partially shed light on the pathogenesis of pregnancy-related complications such as spontaneous abortion and preterm delivery, and provide better strategies to improve perinatal outcomes and offspring’s health^3,4^.

Programmed cell death 1 (PD-1) is well known as an inhibitory transmembrane receptor belonging to the B7-CD28 family and expresses on various activated immune cells, especially activated T cells^5,6^. The engagement of PD-1 and its prior binding ligand programmed death ligand-1 (PDL1) recruits second signals required for T cell exhaustion that are characterized by reduced proliferation, diminished cytokine production, and functionally silenced cytotoxic effector, serving as a negative checkpoint for proper immune responses^7,8^. Several reports have proposed the importance of PD-1-PDL1 interaction involved in pregnancy maintenance, and increased PD-1 expression was detected in decidual immune cells in the first trimester of normal pregnancy^9,10^. In addition, previous studies have established links among PD-1 and PDL1 expression, Th1/Th2/Th17 balance and fetomaternal tolerance^11,12^. A deficiency of PDL1 leads to a reduced ratio of regulatory T/effector T cells (Treg/Teff) and drives a shift toward Th1- and Th17-cell expansion, which represents a barrier to induce tolerance, resulting in increased embryo resorption and miscarriage^11,12^. Our previous study has revealed that the reduction of fetal survival rate induced by PDL1 blockade is associated with enhanced accumulation of T follicular helper (Tfh) cells and upregulated expression of ICOS and PD-1, developing the Th1/Th2/Th17/Treg paradigm into a Th1/Th2/Th17/Treg/Tfh balance in the formation and maintenance of maternal-fetal tolerance during the healthy pregnancy^13^.

As a novel and specialized subset of CD4^+^ T cells, T follicular regulatory (Tfr) cells were first identified in mice as simultaneously expressing Tfh cell-associated molecules (such as CXCR5, ICOS, PD-1, and the transcription factor Bcl6) and Treg cell-characterized proteins (represented by Foxp3)^14–16^. Although sharing nearly identical receptors with Tfh cells, Tfr cells, which are derived from Treg cells exert a potent inhibitory effect that suppress excessive Tfh cell expansion, B cell response and antibody production to orchestrate germinal center (GC) cell dynamics^17–19^. Growing evidence has shown that the dysregulations of Tfr cells including quantity and quality anomalies, as well as the imbalance of Tfh/Tfr cells, are implicated in various immune diseases, such as autoimmunity, chronic inflammatory disease, malignancy, and transplant rejection^19–28^.

However, the role of Tfr cells during pregnancy still remains unelucidated. Here, we introduced an allogeneic normal pregnant mouse model and found that CD4^+^CXCR5^hi^PD-1^hi^Foxp3^+^Tfr cells were preferentially accumulated in the uterus at mid-gestation and displayed a distinct phenotype. In addition, the absence of PDL1 resulted in increased fetal resorption by favoring Tfr cells accumulation and upregulating PD-1 expression on these cells. However, PDL1 blockage altered neither the ratio of Tfh/Tfr cells nor B-cell maturation and differentiation. Collectively, our findings present a correlation of Tfr cells accumulation with healthy allogeneic pregnancy and PDL1 blockade-induced miscarriage, and suggest that Tfr cells may be engaged in the maintenance of maternal-fetal immune tolerance. Furthermore, although blockade of PD-1/PD-L1 checkpoint has been proven to be an effective immunotherapy in some tumors^29–31^, the reproductive safety must be considered because of the increased Tfr cells and fetal losses in PDL1 blocked pregnant mice.

## Materials and methods

### Mice

Pathogen-free female BALB/c (H-2^d^) and male C57BL/6 (H-2^b^) mice (8–10-week-old) were purchased from the Shanghai Laboratory Animal Center, Chinese Academy of Science, and bred in the Department of Laboratory Animal Science, Shanghai Jiaotong University School of Medicine (Shanghai, China). Two adult BALB/c females were co-caged with one C57BL/6 male, and the presence of a vaginal-plug was denoted as embryonic day 0.5 (E0.5). All animal experiments were ethically acceptable and performed in accordance with guidelines for animal care and use in research of Shanghai Jiaotong University School of Medicine (Shanghai, China).

### Preparation of mononuclear cells

Pregnant mice were sacrificed either on E11.5 or E18.5. Isolation and incubation of mononuclear cells from peripheral blood, spleen, thymus, uterus, and bone marrow was conducted as previously described^13,32,33^. In brief, whole blood was harvested from mice via vena orbitalis into phosphate buffered saline (PBS) containing 2% EDTA after intraperitoneal anesthesia with pentobarbital sodium at a dosage of 50 mg/kg. Blood cell suspension was layered by using Lymphoprep^TM^ (AS1114546, Axis-shield) and peripheral blood mononuclear cells (PBMCs) were isolated according to a standard density gradient centrifugation process. The collection of spleen and thymus cells required a syringeplunger to grind the tissue gently and thoroughly, and a 70-μm pore size cell strainer (352350, BD Biosciences) for cell suspensions filtering through. Purified splenic and thymic mononuclear cells were gathered by centrifugation and counted by using a Leica DMI 3000B microscope with trypan blue exclusion after potential contaminating red blood cells being wiped out under the influence of co-incubation with ACK Lysing Buffer (A10492-01, Gibco) for 1 min. To obtain single mononuclear cell suspensions in the uterus, embryos were removed carefully after hysterolaparotomy. The pooled uterus and placentas were finely cut into less than 1 × 1 × 1 mm pieces with ocular scissors, and the minced tissue was then dispersed in PBS and filtered through a 70-μm pore size cell strainer, followed by being purified through density gradient centrifugation with Lymphoprep^TM^. Mice femurs were introduced to isolate mononuclear cells derived from bone marrow, followed by centrifugation to harvest mononuclear cell suspensions.

### Treatment protocol

To induce embryo loss, 250 μg anti-mouse PDL1 blocking monoclonal antibody (mAb) (clone: 10 F.9G2, BioLegend) was administered to each pregnant mouse in experimental group via intraperitoneal (i.p.) injection on E5.5 and E8.5, consecutively. As controls, mice were i.p. treated with the equivalent dosage of IgG at the same gestation day. Both IgG-treated controls and PDL1-blocked pregnant mice were sacrificed on E11.5, and the embryo resorption rate was calculated as: Resorption rate (%) = [number of resorbed embryos/number of total (resorbed + viable) embryos] × 100.

### mAbs and reagents

Fluorescein-conjugated anti-mouse mAbs, including anti-CD4-Pacific Blue (clone: RM4-5), anti-CD8-V500 (53-6.7), anti-CXCR5-PE-Cy7 (2G8), anti-CD95 (FAS)-PE-Cy7 (Jo2), and anti-CD138-APC (281-2) were purchased from BD Pharmingen; anti-FOXP3-PE-Cyanine 5 (FJK-16s) and anti-GL7-Alexa Fluor 488 (GL-7) were from eBioscience; and anti-CD19-PE (6D5), anti-CD279 (PD-1)-PE (RMP1-30), anti-BCL-6-APC (7D1), and anti-IgG (minimal x-reactivity)-PerCP-Cy5.5 (Poly4053) were from BioLegend.

### Flow cytometry

Surface and intranuclear stainings were carried out by multicolor flow cytometry (FCM) as we and others described previously^34–37^. For cell surface staining, freshly isolated mononuclear cells were resuspended in 100 μL PBS containing 3% (v/v) fetal bovine serum (FBS) with different fluorescein-conjugated mAbs. After incubation at room temperature in the dark for 30 min, the labeled cells were washed with PBS twice and collected for later use. Intranuclear staining of Foxp3 and BCL-6 was performed in line with the instruction for Foxp3 staining (00-5523-00, eBioscience) after surface combination. Cells were fixed and permeabilized using Fixation/Permeabilization Buffer for 1 h, followed by being incubated with directly conjugated specific antibody for 40 min at room temperature. Finally, we used a BD FACS Canto II flow cytometer (BD Biosciences, USA) to collect the immunostained cells and the FlowJo 7.6.1 software to analyze the data.

### Statistical analyses

Statistical analyses were implemented in Graphpad Prism 5 software and the results were shown as mean ± standard error of means (M. ± S.E.M.). The normality of our data was evaluated by the Shapiro-Wilk normality test. If the data followed normal distribution, we performed statistical analysis with unpaired or paired Student’s *t*-test, or one-way ANOVA with subsequent Tukey’s post-tests; if not, we analyzed the data by using the Mann–Whitney *U* or Wilcoxon matched pairs test, or Kruskal-Wallis test followed by Dunns multiple-comparison test. *P*-values < 0.05 were considered to be significant difference. Details are described in the Figure Legends. Randomization, blinding, and sample size estimation tests were not done for our animal studies.

## Results

### Result 1: CD4^+^CXCR5^hi^PD-1^hi^Foxp3^+^Tfr cells are preferentially accumulated in the uterus at mid-gestation

**Fig. 1:**
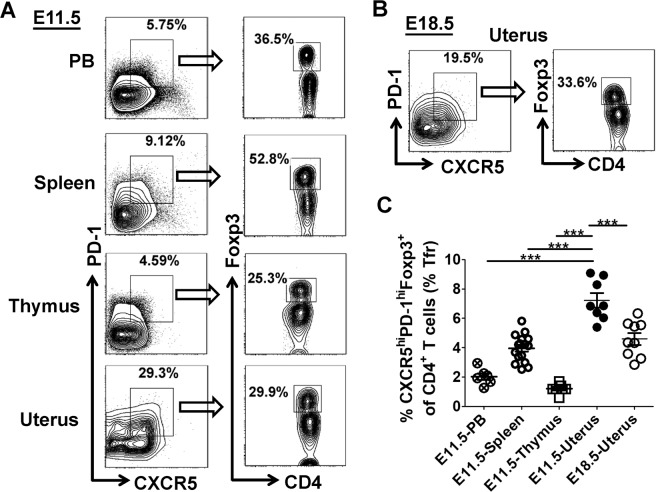
CD4^+^CXCR5^hi^PD-1^hi^Foxp3^+^ Tfr cells are preferentially accumulated in the uterus at mid-gestation. Adult female BALB/c and male C57BL/6 mice were co-caged in a 2:1 ratio and the presence of a vaginal-plug was taken for embryonic day 0.5 (E0.5). **a**–**c** Representative flow cytometric plots (**a**, **b**) and cumulative data (**c**) illustrating the percentage of CXCR5^hi^PD-1^hi^Foxp3^+^ population gated in CD4^+^ T cells derived from the PB, spleen, thymus and uterus of maternal mice on E11.5 (**a**, **c**) as well as from the uterus on E18.5 (**b**, **c**). Each symbol reflects the data from a single mouse (*n* ≥ 7 mice per group) and the data are representative of two independent experiments. The cells are gated in CD4^+^ T cells. Data were assessed statistically using one-way ANOVA followed by Tukey’s multiple-comparison test. PB peripheral blood; hi high; ***p < 0.001.

Tfr cells have been usually defined as the CXCR5^hi^PD-1^hi^Foxp3^+^ population gated in CD4^+^ T cells^19,22^. To identify whether Tfr cells are involved in immune homeostasis at the maternal–fetal interface during allogeneic-normal pregnancy, adult BALB/c females were mated with C57BL/6 males and the presence of a vaginal-plug was taken for embryonic day 0.5 (E0.5). We performed multi-color flow cytometry (FCM) to screen CD4^+^CXCR5^hi^PD-1^hi^Foxp3^+^ Tfr cells among different lymphoid tissues, and found that the proportion of these cells was remarkably higher in the uterus than that in the peripheral blood (PB), spleen and thymus on E11.5 (Fig. 1a, c). Moreover, the proportion of CD4^+^CXCR5^hi^PD-1^hi^Foxp3^+^ Tfr cells in the uterus was significantly reduced on E18.5 than E11.5 (Fig. 1b, c), but very few CD4^+^CXCR5^hi^PD-1^hi^Foxp3^+^ Tfr cells were detected in the uterus on E5.5-7.5 (Supplementary Fig. 1). However, although the absolute number of splenic CD4^+^CXCR5^hi^PD-1^hi^Foxp3^+^ Tfr cells dramatically increased on E11.5 and then returned to the normal level on E18.5, the proportion of these cells was not changed in the spleen during pregnancy (Supplementary Fig. 2). Taken together, these data indicate that CD4^+^CXCR5^hi^PD-1^hi^Foxp3^+^ Tfr cells are dominantly enriched in the uterus at mid-gestation.

### Result 2: uterine Tfr cells display a distinct phenotype

**Fig. 2:**
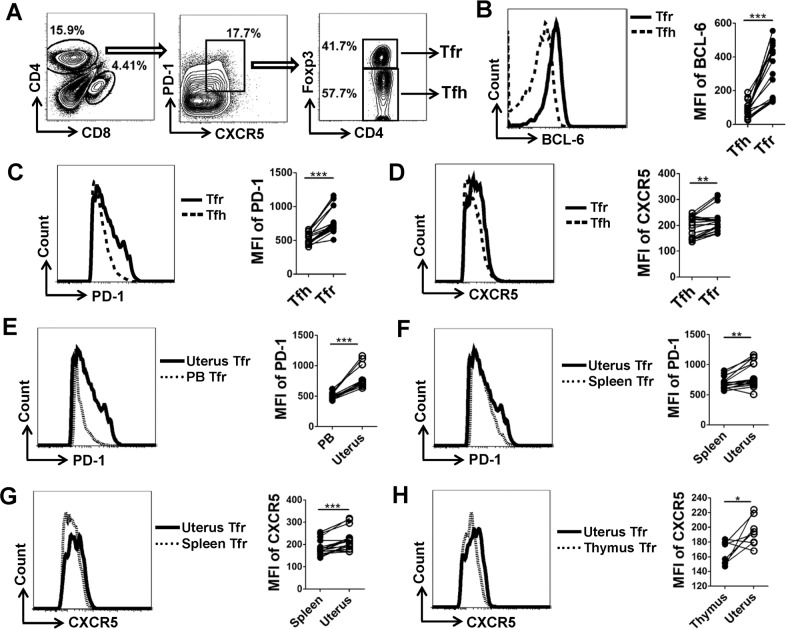
Uterine Tfr cells display a distinct phenotype. **a** CD4^+^ T cells are gated from the lymphocytes in the uterus of pregnant mice on E11.5, and a flow diagram illustrates the method used to distinguish CD4^+^CXCR5^hi^PD-1^hi^Foxp3^−^ Tfh and CD4^+^CXCR5^hi^PD-1^hi^Foxp3^+^ Tfr cells. **b**–**d** Intra-nuclear expression of BCL-6 was detected according to the Foxp3 staining protocol. Representative flow cytometric histograms and cumulative data illustrate the comparison of BCL-6, PD-1 and CXCR5 expression between uterine Tfr and Tfh cells. **e**–**h** Representative flow cytometric histograms and cumulative data compare the PD-1 expression on Tfr cells derived from the PB, spleen and uterus (**e**, **f**), as well as the CXCR5 expression on Tfr cells derived from the spleen, thymus and uterus (**g**, **h**), of maternal mice on E11.5. Each symbol reflects the data from a single mouse (*n* ≥ 6 mice per group). Geometric MFI values were calculated using FlowJo 7.6.1 software and the data were assessed statistically using Wilcoxon matched pairs test. PB peripheral blood; MFI mean fluorescent intensity; *p < 0.05; **p < 0.01; ***p < 0.001.

As previous studies have demonstrated, Tfr cells not only express similar surface antigens and transcription factors to Tfh cells including CXCR5, PD-1, ICOS, and BCL-6, but also are characterized by typical Treg cell markers such as Foxp3, and Tfr and Tfh cells can be distinguished based on Foxp3 expression^14,19^. Similarly, we identified Tfr cells as CXCR5^hi^PD-1^hi^Foxp3^+^ population and Tfh cells as CXCR5^hi^PD-1^hi^Foxp3^−^ population, both of which were gated in CD4^+^ T cells (Fig. 2a).

To characterize the Tfr cells residing at the uterus, FCM was implemented to determine the expression of characteristic proteins at mid-gestation. Surprisingly, the level of BCL-6, PD-1 and CXCR5 was notably higher in uterine Tfr than Tfh cells (Fig. 2b–d). In addition, uterine Tfr cells presented a higher expression of PD-1 as compared with that in the PB and spleen, as well as a greater frequency of CXCR5 than that in the spleen and thymus (Fig. 2e–h). However, Tfr cells residing in the uterus and other lymphoid tissues displayed no palpable difference in the expression of BCL-6 and Foxp3 (Supplementary Fig. 3). These results verify that uterine Tfr cells display a distinct phenotype as compared with those in other lymphoid tissues as well as uterine Tfh cells.

### Result 3: PDL1 blockage increases fetal resorption and Tfr cells

**Fig. 3:**
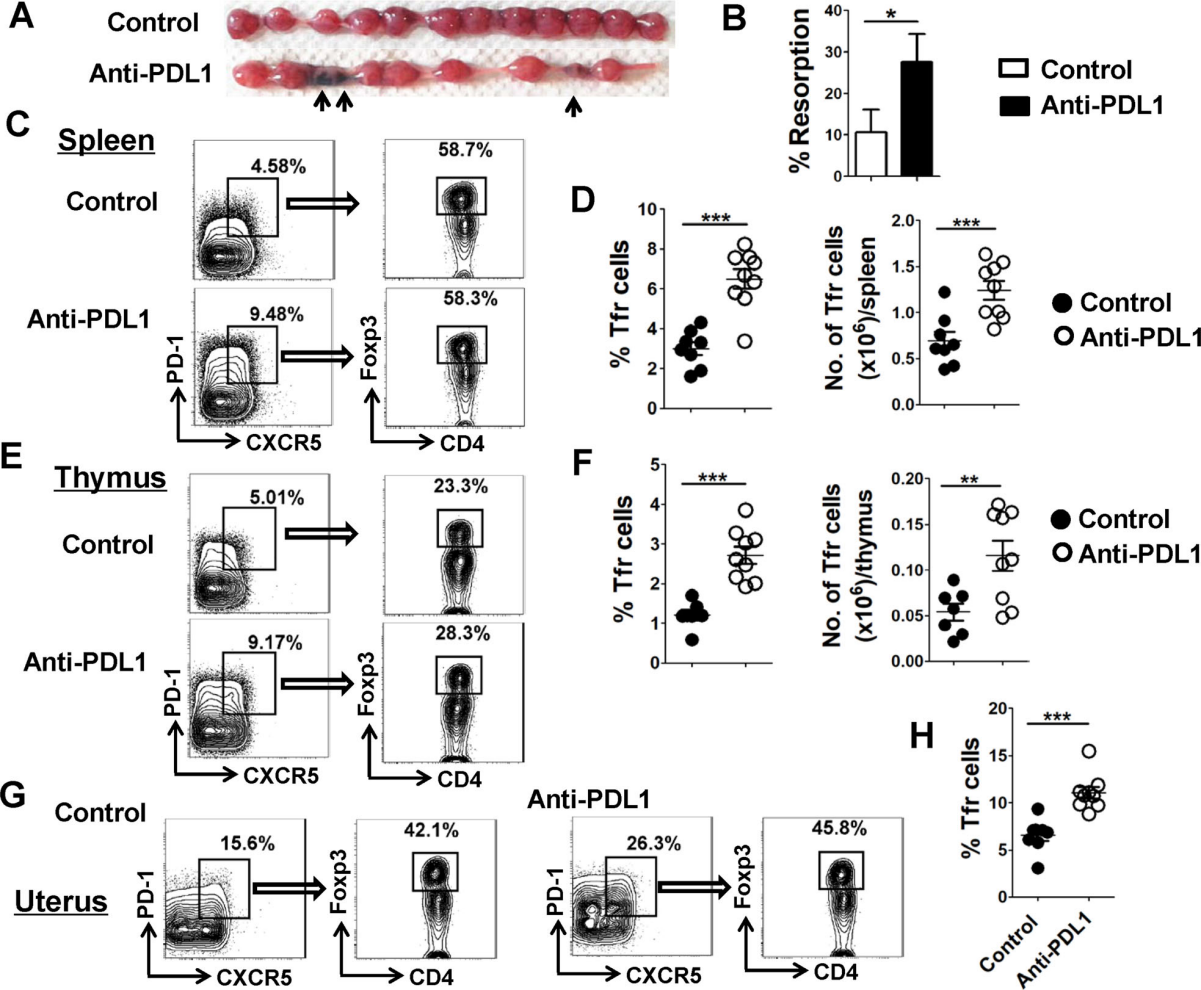
PDL1 blockage increases fetal resorption and Tfr cells. Adult BALB/c females were mated with C57BL/6 males, and the pregnant mice were injected intraperitoneally with anti-mouse PDL1 blocking mAb at a dose of 250 μg on E5.5 and E8.5, respectively. Both the control (IgG injected) and PDL1-blocked mice were sacrificed on E11.5. **a** Representative diagrams illustrate the whole uterus (containing embryos and placentas) and resorbed embryos are indicated by arrows. **b** Resorption rate is compared between the control and PDL1-blocked mice. **c**–**h** Representative flow cytometric plots (**c**, **e**, **g**) and cumulative data (**d**, **f**, **h**) compare the percentage and absolute number of CD4^+^CXCR5^hi^PD-1^hi^Foxp3^+^ Tfr cells in the maternal spleen (**c**, **d**), thymus (**e**, **f**) and uterus (**g**, **h**) between the control and PDL1-blocked mice. Each symbol reflects the data from a single mouse (*n* ≥ 7 mice per group) and the data are combined from four independent experiments. The cells are gated from CD4^+^ T cells. The data were assessed statistically using unpaired Student’s *t*-test (**b**, **d**) or Mann–Whitney *U* test (**f**, **h**). No.: number; *p < 0.05; **p < 0.01; ***p < 0.001.

To induce an abortion mouse model, pregnant BALB/c females, which had been mated with C57BL/6 males, were administrated intraperitoneally with anti-PDL1 blocking monoclonal antibody (mAb) or IgG (as a nonspecific control) at a dosage of 250 μg on E5.5 and E8.5, respectively. Mice with both treatments were sacrificed on E11.5. The whole uterus was separated and more embryo losses were visibly observed in the PDL1-blocked group (Fig. 3a, b), which was consistent with previous reports^12,13^. Furthermore, the proportion as well as the absolute number of CD4^+^CXCR5^hi^PD-1^hi^Foxp3^+^ Tfr cells in the spleen, thymus, and uterus exhibited an apparent increase after the administration of anti-PDL1 mAb (Fig. 3c–h and data not shown), suggesting that the higher rate of fetal resorption due to PDL1 blockade may be associated with the enhanced accumulation of Tfr cells.

### Result 4: PDL1 blockage upregulates PD-1 expression on Tfr cells

**Fig. 4:**
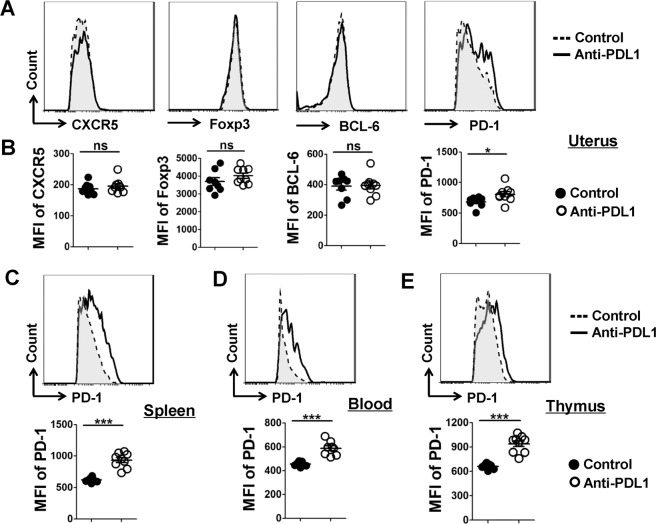
PDL1 blockage upregulates PD-1 expression on Tfr cells. **a**, **b** Representative flow cytometric histograms and cumulative data compare the molecular expression of CXCR5, Foxp3, BCL-6 and PD-1 on/in uterine Tfr cells between the control and PDL1-blocked mice. **c**–**e** Representative flow cytometric histograms and cumulative data illustrating the expression level of PD-1 on Tfr cells derived from the spleen (**c**), blood (**d**), and thymus (**e**) of the control and PDL1-blocked mice. Each symbol reflects the data from a single mouse (*n* ≥ 5 mice per group). Cells are gated in CD4^+^CXCR5^hi^PD-1^hi^Foxp3^+^Tfr cells. Geometric MFI values were calculated using FlowJo 7.6.1 software and the data were assessed statistically using unpaired Student’s *t*-test. MFI mean fluorescent intensity; ns not significant; *p < 0.05; ***p < 0.001.

Next, we explored whether the expression of characteristic molecules on Tfr cells was also altered after treating pregnant mice with anti-PDL1 mAb. Our results demonstrated that the expression of PD-1 on CD4^+^CXCR5^hi^PD-1^hi^Foxp3^+^Tfr cells from PDL1-blocked mice was dramatically higher than that from the controls in the uterus, spleen, PB, and thymus, while the expression of other characteristic proteins including CXCR5, BCL-6, and Foxp3 on/in Tfr cells displayed no significant difference between anti-PDL1treatment and control groups in the uterus and other lymphoid tissues (Fig. 4 and Supplementary Fig. 4). These data suggest that the increased fetal resorption resulted from PDL1 absence may correlate with the high expression of PD-1 on Tfr cells.

### Result 5: PDL1 blockage does not affect the ratio of Tfh/Tfr cells

**Fig. 5:**
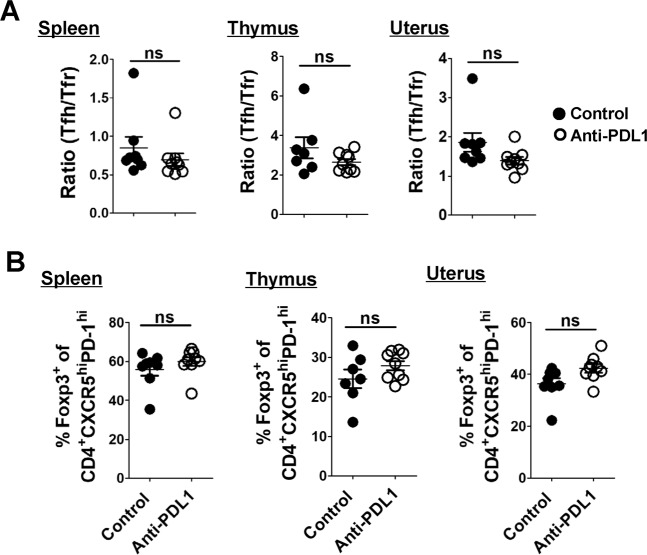
PDL1 blockage does not affect the ratio of Tfh/Tfr cells. **a**, **b** Cumulative data comparing the ratio of Tfh/Tfr cells (**a**) as well as the percentage of Foxp3^+^ population among CD4^+^CXCR5^hi^PD-1^hi^ T cells (**b**) derived from the spleen, thymus, and uterus between the control and PDL1-blocked mice on E11.5. Each symbol reflects the data from a single mouse (*n* ≥ 7 mice per group) and the data are combined from four independent experiments. The data were assessed statistically using Mann–Whitney *U* test. ns not significant.

Tfh cells are essential for GC formation and maintenance, and provide survival signals for high affinity B cells within the GC for their differentiation to isotype-switched antibody secreting cells and memory B cells^38,39^. Although the function of Tfr cells in the GC has not been completely clarified, the mainstream view currently is that Tfr cells restrict Tfh- and B-cell proliferation and exert an inhibitory action on antibody production^40–42^. The ratio of Tfh/Tfr cells plays a crucial role in humoral immunity and regulates antibody responses, and effective humoral immunity depends on the delicate balance between promotive Tfh cells and suppressive Tfr cells^28,43^. Our previous study^13^ and above data (Fig. 3) showed that PDL1 blockage increased both Tfh and Tfr cells simultaneously. Hence, the ratio of Tfh/Tfr cells was further examined, and we found that the ratio showed no statistical difference between PDL1-blocked and control mice, accompanied by no difference in the proportion of Foxp3^+^ cells among CD4^+^CXCR5^hi^PD-1^hi^ population between these mice (Fig. 5).

### Result 6: PDL1 blockage does not affect B-cell maturation and differentiation

**Fig. 6:**
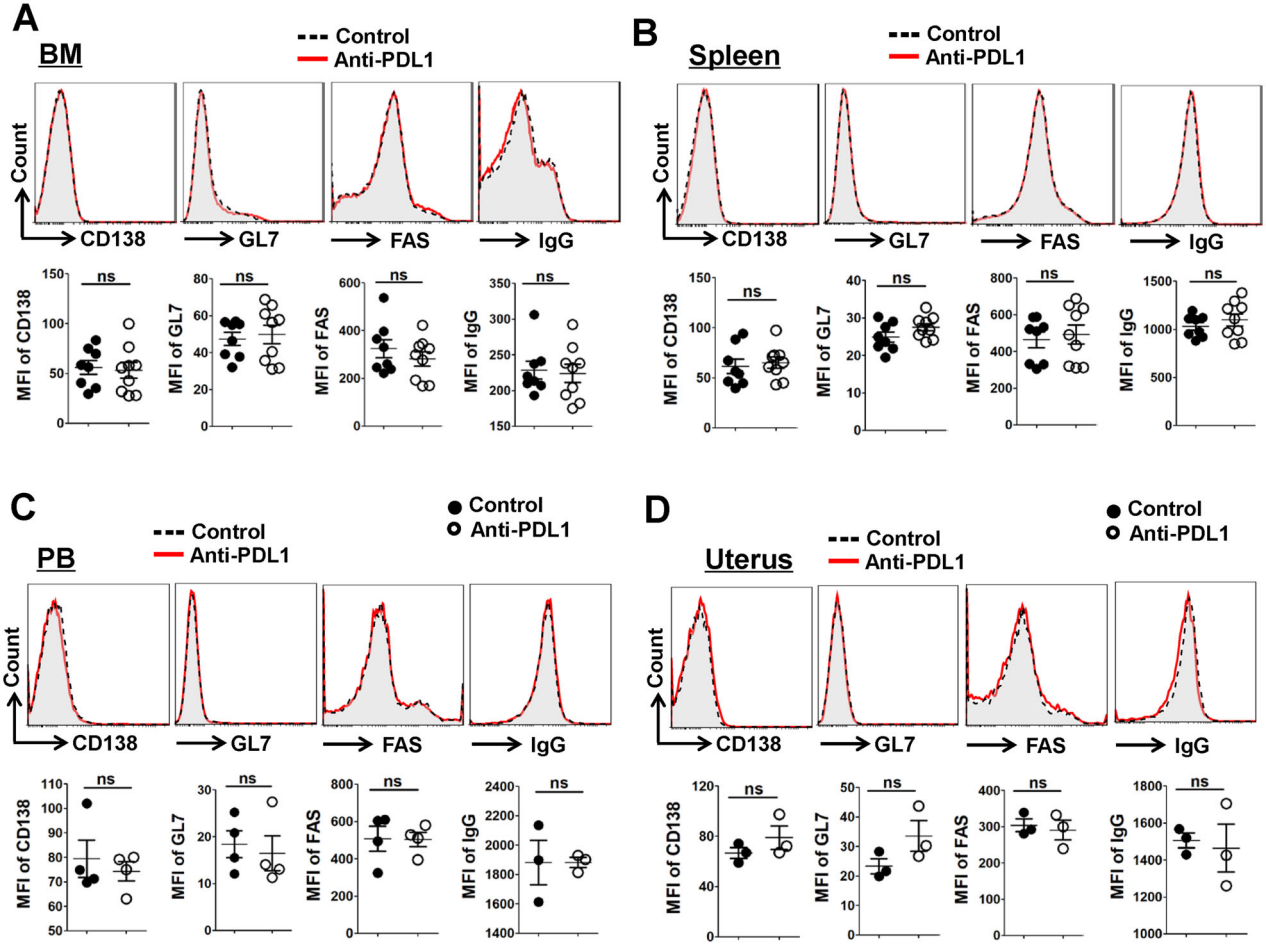
PDL1 blockage does not affect the expression of GC-resident B cell markers. **a**–**d** Representative flow cytometric histograms and cumulative data illustrating the molecular expression of CD138, GL7, FAS, IgG on CD19^+^ B cells derived from the BM (**a**), spleen (**b**), PB (**c**), and uterus (**d**) of the control and PDL1-blocked mice. Cells are gated in B cells and each symbol reflects the data from a single mouse (*n* ≥ 3 mice per group). Cells are gated in CD19^+^ B cells. Geometric MFI values were calculated using FlowJo 7.6.1 software and the data were assessed statistically using Mann–Whitney *U* test. MFI mean fluorescent intensity; BM bone marrow; PB peripheral blood; ns not significant.

**Fig. 7:**
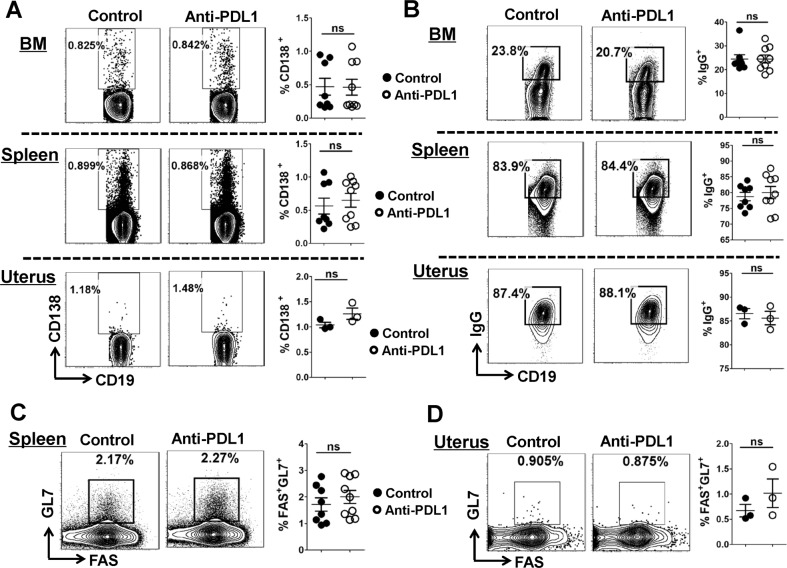
PDL1 blockage does not affect the proportion of CD138^+^ plasma cells, IgG^+^ antibody-producing B cells, and GL7^+^ FAS^+^GC B cells. **a–d** Representative flow cytometric plots and cumulative data illustrating the proportion of CD138^+^ plasma cells (**a**), IgG^+^ antibody-producing B cells (**b**), and GL7^+^FAS^+^GC B cells (**c**, **d**) in the BM, spleen or uterus of the control and PDL1-blocked mice. Cells are gated in CD19^+^ B cells. Each symbol reflects the data from a single mouse (*n* ≥ 3 mice per group) and the data were assessed statistically using Mann–Whitney *U* test. BM bone marrow; ns not significant.

Given the inhibitory effect of Tfr cells, finally, we asked whether PDL1 blockade impact B-cell maturation and differentiation. However, the proportion of CD19^+^ total B cells; the expression of GC-resident B cell markers including CD138, GL7, FAS, and IgG; together with the proportion of CD138^+^ plasma cells, IgG^+^ antibody-producing B cells and GL7^+^ FAS^+^GC B cells; were not apparently different between the control and PDL1-blocked mice in the bone marrow (BM), spleen, PB and uterus (Figs. 6 and 7 and Supplementary Figs. 5 and 6). Above all, there is a paucity of convincing evidence concerning the role of PDL1 blockade in the maturation and differentiation of B cells.

## Discussion

Multiple mechanisms, the redistribution of immune cells in particular, contribute to the elaborate balance of immune clearance and immune tolerance at the maternal-fetal interface. Putative CD4^+^ effector T helper (Th) cells Th1⁄Th2⁄Th17 and Treg paradigms gave their importance in fetal graft rejection and adoption, and either bias may result in different kinds of pregnancy failures including spontaneous abortion, preterm delivery, pre-eclampsia, fetal growth restriction, and even death^44–46^.

The newly concerned Tfr cells have attracted much interest due to their unique roles in immunoregulation. Previous studies have demonstrated that this subpopulation of CD4^+^ T cells co-opts Tfh differentiation pathway and upregulates the transcriptional repressor Bcl-6 which is required for the generation of Tfh cells and the suppression of Th1, Th2, and Th17 cell differentiation^15,47,48^. Recently, we proposed Tfh cells as a crucial player involved in the gestation, enriching the original paradigm into a novel Th1/Th2/Th17/Treg/Tfh paradigm^13^. Our data showed that Tfh cells were clustered in the uterus at mid-gestation, however, PDL1-blockade-induced excessive abundance of Tfh cells might step back the development and lead to embryo loss^13^. Since Tfh and Tfr cells share a similar phenotype but an opposite function, we next investigated the characteristics and roles of Tfr cells during pregnancy.

Herein, we defined Tfr cells as CXCR5^hi^PD-1^hi^Foxp3^+^ population gated in CD4^+^ T cells as previous studies performed^19,22^. Interestingly, we found that CD4^+^CXCR5^hi^PD-1^hi^Foxp3^+^ Tfr cells were more prone to enriched in the uterus at mid-gestation (Fig. 1), and the proportion of splenic CD4^+^CXCR5^hi^PD-1^hi^Foxp3^+^ Tfr cells was not significantly changed after gestation (Supplementary Fig. 2). These data confirmed a redistribution of Tfr cells during normal pregnancy. Furthermore, human decidual tissues and peripheral blood samples were collected from healthy women who were undergoing early elective abortions in the first trimester of pregnancy (at 6–12 weeks of gestation), and flow cytometry analysis showed that the proportion of CD4^+^CXCR5^hi^PD-1^hi^Foxp3^+^Tfr cells was significantly higher in human deciduas than that in peripheral blood (Supplementary Fig. 7). These data were consistent with our observation in the allogeneic pregnancy mouse model (Fig. 1).

An earlier RNA-seq data demonstrated that Tfr cells showed a peculiarly higher expression of CXCR5, and another publication revealed that Tfr cells displayed a greater frequency of PD-1 than Tfh cells^49,50^. Almost coherently, we found that the level of BCL-6, PD-1 and CXCR5 was notably higher in uterine Tfr than Tfh cells (Fig. 2a–d). Previous studies have shown that PDL1 is highly expressed on the fetal extravillous trophoblast cells (EVTs) and decidual stroma cells (DSCs) during human and mouse early pregnancies^4,51,52^, and DSCs can secrete C-X-C motif chemokine 13 (CXCL13), which may contribute to a high level of this chemokine detected in the amniotic fluid of pregnant women^53,54^. Therefore, we speculate that DSCs/EVTs-derived PDL1 (a prior binding ligand of PD-1) and CXCL13 (the only known ligand for CXCR5) facilitate the recruitment and retention of Tfr cells with a high expression of PD-1 and CXCR5 in the uterus, which contribute to our observation that uterine Tfr cells display a distinct phenotype as compared with those in other lymphoid tissues (Fig. 2e–h).

Despite antibody-mediated rejection (AMR) is dominantly attributed to B cells maturation and specific antibody production, T cells are considered as principle infiltration cells in the respect of rejection^55^. Tfr cells are believed to induce the inability of Tfh cells in the GC, specifically preventing incessant antibody-produced B cell responses. Subsequent evidence suggested that Tfr cells effectively regulated humoral immune responses in autoimmune diseases and organ transplantation^19,56–59^. A leading theory is that Tfr cells are formidable adversaries against chronic graft-versus-host diseases (cGVHD)^59,60^. In patients with chronic renal allograft rejection, the number and the ratio of Tfr cells were significantly attenuated compared with non-AMR patients, while IL-21-producing Tfh cells were obviously augmented, indicating that Tfr cells can remove barriers to facilitate a successful transplant^58^. The semi-allogeneic fetus is long regarded as a natural transplantation model in vivo, that allograft rejection may lead to miscarriage or other pregnancy complications^61^. Consistent with the mainstream findings in other transplantations^58,62,63^, our results showed a higher proportion of CD4^+^CXCR5^hi^PD-1^hi^Foxp3^+^ Tfr cells infiltrated in the uterus (Fig. 1), indicating that Tfr cells may participate in maintaining the harmony between the allogeneic fetus and the matrix.

PD-1 is a proverbial inhibitory receptor expressed by T cells, B cells, monocytes, and dendritic cells (DCs), and B cells and DCs express its ligands, PDL1 and PDL2^64^. The PD-1–PDL1 signaling pathway has been widely regarded as a major contributor in maintaining fetomaternal immune tolerance. First, Indira Guleria, et al. found that blockade of PDL1 signaling resulted in increased fetal rejection during murine allognenic pregnancy but not syngeneic pregnancy, and this rejection depended on T cells but not B cells and APCs by using RAG-1-deficient and B cell-deficient mice^52^. Subsequently, they demonstrated that PDL1 blockade led to an expansion of both Th1 and Th17 cells but a suppression of Treg and Th2 cells^10,12,52^. Recently, we proposed Tfh cells as a key player involved in the gestation and found that the increased foetal resorption by PDL1 blockade was accompanied by enhanced accumulation of Tfh cells^13^. In this study, our data showed that PDL1 blockage increased CD4^+^CXCR5^hi^PD-1^hi^Foxp3^+^ Tfr cells but did not affect B-cell maturation and differentiation, and our study is the first report using an allognenic model pregnancy to establish a link among PD-1–PDL1 signaling pathway, Tfr cells, and fetomaternal immune tolerance. Therefore, our data together with previous studies indicated that PD-1–PDL1 signaling pathway plays a key role in maintaining fetomaternal tolerance by favoring the expansion of Th1, Th17, Tfh and Tfr cells but limiting the expansion of Th2 and Treg cells.

Interestingly, PD-1 expression on Tfr cells was observed to be significantly upregulated in PDL1-deficient mice (Fig. 4), and we proposed two possible explanations for this finding. (1) Although the role of PD-1/PDL1 pathway in T cell exhaustion has attracted a great deal of attention, PD-1 is not considered as an exhaustion-specific marker^5,65^. PD-1 is also highly expressed by all T cells during activation as a natural brake to fine-tune the T cell responses, which is an important regulatory manner in normal host physiology^5^. In this study, we used anti-PDL1 blocking antibody to disturb the interaction between PD-1 and PDL1. The PD-1 signaling in Tfr cells was interfered for lacking engagement of the ligand PDL1, which might result in the activation of Tfr cells accompanied by the upregulated expression of PD-1 on these cells. (2) Critical lack of PD-1/PDL1 interaction by blocking PDL1 promoted Tfr cells to express more PD-1 to seek for more effective combination between PD-1 and PDL1, and to receive more signals provided by the ligand PDL1. Overall, several reports have shown that PDL1 blockade leads to an increase in the number and function of Tfr cells^13,20,50^. However, we hold the opinion that the surface receptor PD-1 cannot deliver inhibitory signals without following its engagement with PDL1, and it is the attenuation of PD-1 signaling rather than the augmented expression of PD-1 that contribute to the greater abundance and enhanced capability of Tfr cells.

Successful humoral immunity as well as B cell immunity depends on the intricate balance between stimulatory Tfh cells and inhibitory Tfr cells^18^. Sage et al. revealed that the PD-1/PDL1 pathway controls not only the generation of Tfr cells but also the ratio of Tfh to Tfr cells in the lymph nodes and blood^50^. As Tfr cells exert an inhibitory role in restricting the over-abundance of Tfh and B cells, as well as suppressing the antibody-producing B cell responses in the GC^40–42^, we wondered if PDL1 blockade-related Tfr cells accumulation had an impact on Tfh/Tfr cells ratio and B cells maturation. Surprisingly, we found that neither the ratio of Tfh/Tfr cells nor the proportion of Foxp3^+^ cells among CD4^+^CXCR5h^i^PD-1^hi^ population in the spleen, thymus and uterus was altered in a statistically significant manner (Fig. 5). Beyond that, PDL1 deficiency appeared no impact on B-cell maturation and differentiation in the BM, spleen, PB or uterus (Figs. 6 and 7 and Supplementary Figs. 5 and 6).

Consistent with our findings that PDL1 blockage increases both Tfh^13^ and Tfr cells (Fig. 3), growing evidence has shown that the deficiency of PD-1 or PD-L1 results in more Tfh cells and/or Tfr cells^20,50,66^. However, some studies have shown that the humoral responses are enhanced^67–69^ while others found that they are weakened^70–72^ after blocking PD-1/PDL1 signaling. In this study, we demonstrated that PDL1 blockade affected neither the ratio of Tfh/Tfr cells nor the maturation and differentiation of B cells. Despite the opposing roles of Tfh and Tfr cells on humoral responses, PD-1 is highly expressed on both of them^73^. In addition, the PD-1/PDL1 signaling exerts inhibitory effects on their proliferation and differentiation, and both Tfh and Tfr cells expand when the pathway is prevented^50,74^. Regarding the divergent findings about the effects of PD-1/PDL1 blockage on humoral responses, we are inclined of the view that PD-1/PDL1 signaling may play a context-dependent role in humoral immune responses^20^.

Besides the old concept that B cells participate in fetal-maternal immune tolerance via attenuating B-cell capabilities characterized by diminished immune responses and reduced auto-antibodies, recent studies have demonstrated that the increased regulatory B cells (Bregs) play an indispensable role in preventing semi-allogeneic rejection and promoting a stable tolerant microenvironment during normal pregnancy^75–78^. Of particular note, Tfr cells not only regulate GC reaction by inhibiting B-cell maturation, differentiation and antibody production, but also provide IL-10 for Breg-cell proliferation and differentiation^79,80^. Our findings showed that Tfr cells were consumingly accumulated in the uterus during early-mid gestation. Thus, we speculated that these Tfr cells could limit excessive auto-antibodies produced by B cells, and stimulate the generation of Bregs by providing IL-10, which contributed to the maintenance of normal pregnancy. As mentioned above, previous reports have revealed that blockade of PDL1 signaling results in increased fetal rejection during murine allognenic pregnancy but not syngeneic pregnancy, and this rejection depends on T cells but not B cells and APCs^52^. In addition, deficiency of PDL1 leads to an expansion of Th1, Th17 and Tfh cells but a suppression of Treg and Th2 cells^10,12,13,52^. In this study, we found PDL1 blockage increased fetal resorption and Tfr cells. Nevertheless, we consider the augmented fetal resorption rate and the disordered immune tolerance status not only as a consequence of Tfr cell expansion, but also as a result of the imbalanced Th cell differentiation: in favor of Th1, Th17, and Tfh cells while against the development of Th2 and Treg cells. Overall, we proposed a new Th1/Th2/Th17/Treg/Tfh/Tfr paradigm during a healthy pregnancy that any of the participants play an important role in maintaining the immune tolerance at fetal-maternal interface during healthy pregnancy.

In summary, we have first presented a previously unknown correlation of Tfr cells accumulation with healthy allogeneic pregnancy and PDL1 blockade-induced miscarriage. We demonstrate that CD4^+^CXCR5^hi^PD-1^hi^Foxp3^+^ Tfr cells are preferentially enriched in the uterus at mid-gestation and display a distinct phenotype. In addition, increased fetal resorption induced by PDL1 absence has a strong correlation with excessive Tfr cells infiltration and upregulated PD-1 expression on these cells. However, there is no significant change in the Tfh/Tfr ratio as well as the maturation and differentiation of B cells after PDL1 blockade. Collectively, these findings update our knowledge of the orchestrated immunological alternations during normal pregnancy and provide insightful ideas into the pathogenesis of abortion. Moreover, our data indicate that although antibodies that block PD-1/PDL1 checkpoint pathway including anti-PD-1 and anti-PDL1 are proving to be an effective immunotherapy in some tumors with striking clinical trial results^29–31^, the reproductive safety must be considered when these are used in pregnancy because of the increased inhibitory Tfr cells and fetal losses in PDL1 blockade mice.

## Supplementary information

Supplemental Figure 1

Supplemental Figure 2

Supplemental Figure 3

Supplemental Figure 4

Supplemental Figure 5

Supplemental Figure 6

Supplemental Figure 7

Supplementary Figure Legend

Supplemental Material and Method
