# Supplementary figures and images for: PDL1 blockage increases fetal resorption and Tfr cells but does not affect Tfh/Tfr ratio and B-cell maturation during allogeneic pregnancy
Source: Cell Death Dis. 2020 Feb 12;11(2):119. doi: 10.1038/s41419-020-2313-7 (PMC7016117; doi:10.1038/s41419-020-2313-7)

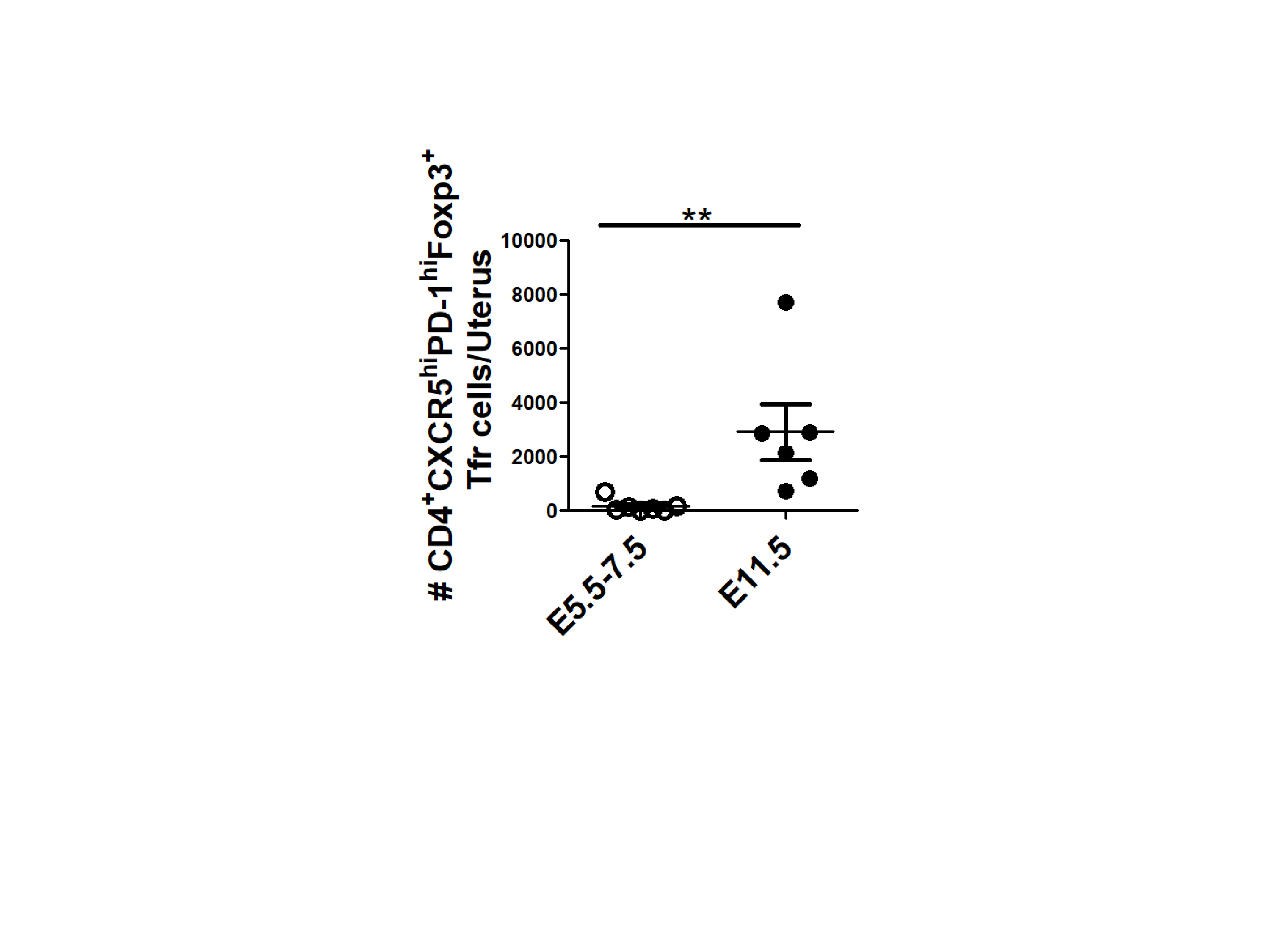

Supplement: Supplementary file 1 — Supplemental Figure 1 [file 41419_2020_2313_MOESM1_ESM.tif]

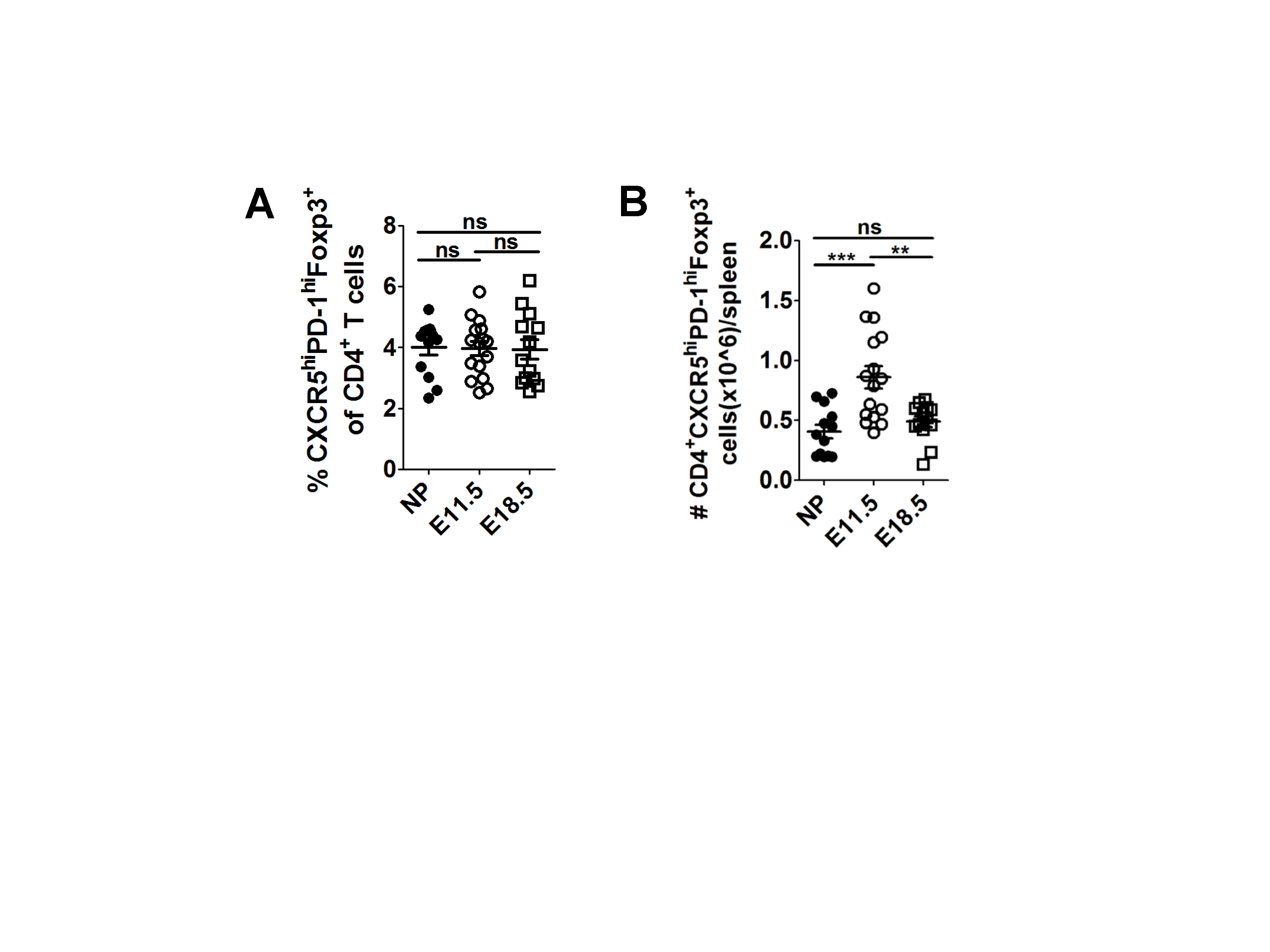

Supplement: Supplementary file 2 — Supplemental Figure 2 [file 41419_2020_2313_MOESM2_ESM.tif]

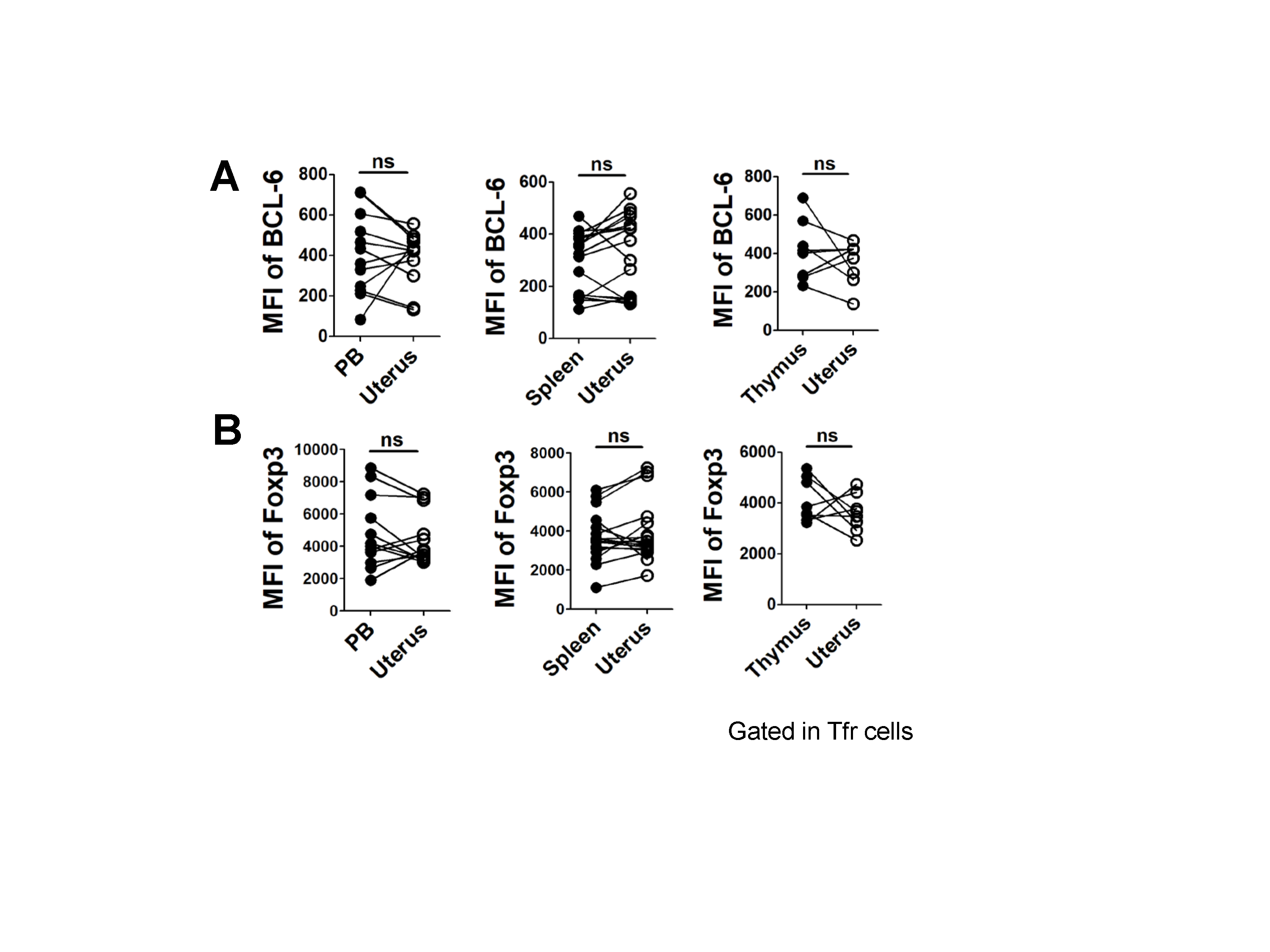

Supplement: Supplementary file 3 — Supplemental Figure 3 [file 41419_2020_2313_MOESM3_ESM.tif]

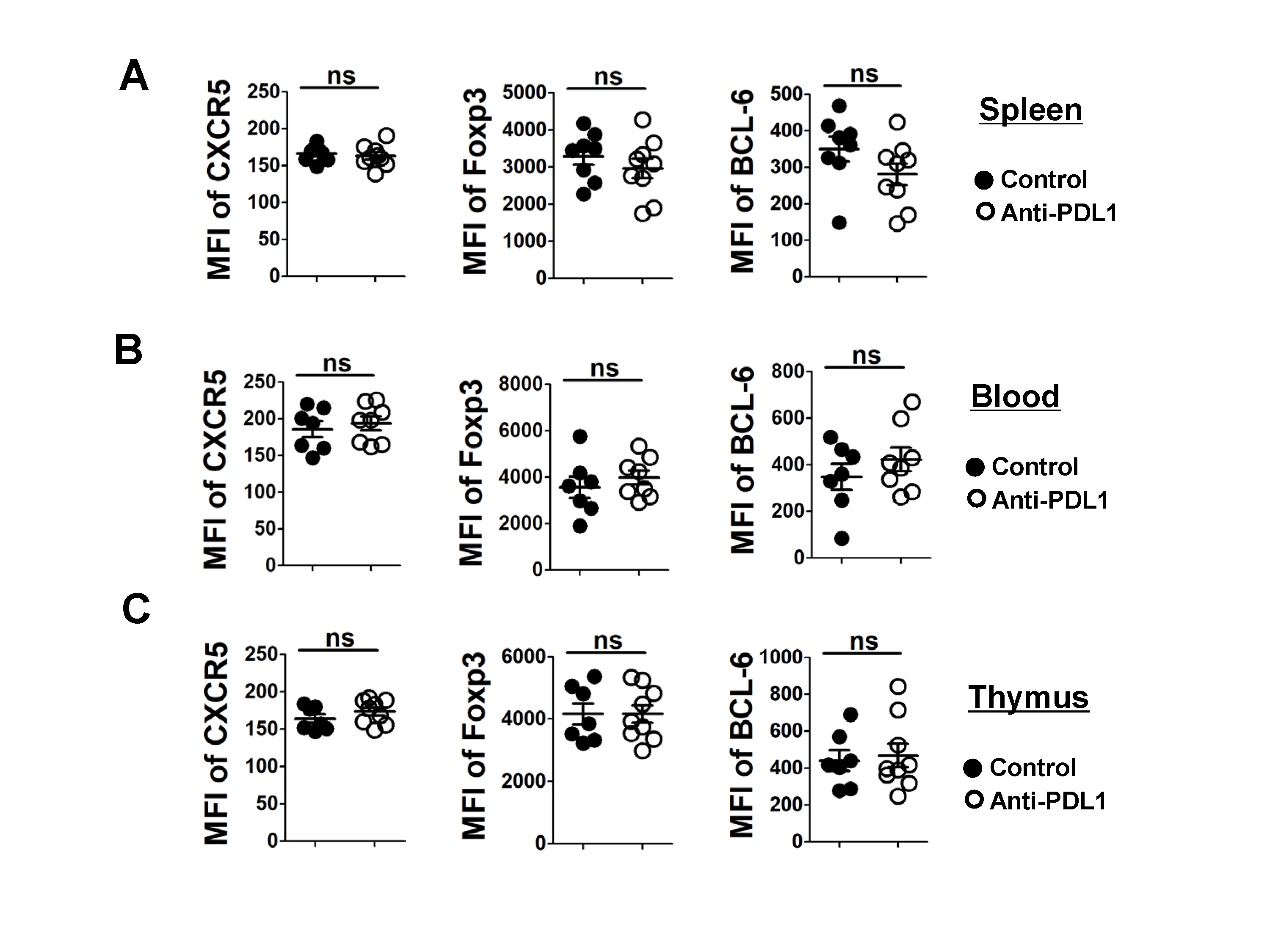

Supplement: Supplementary file 4 — Supplemental Figure 4 [file 41419_2020_2313_MOESM4_ESM.tif]

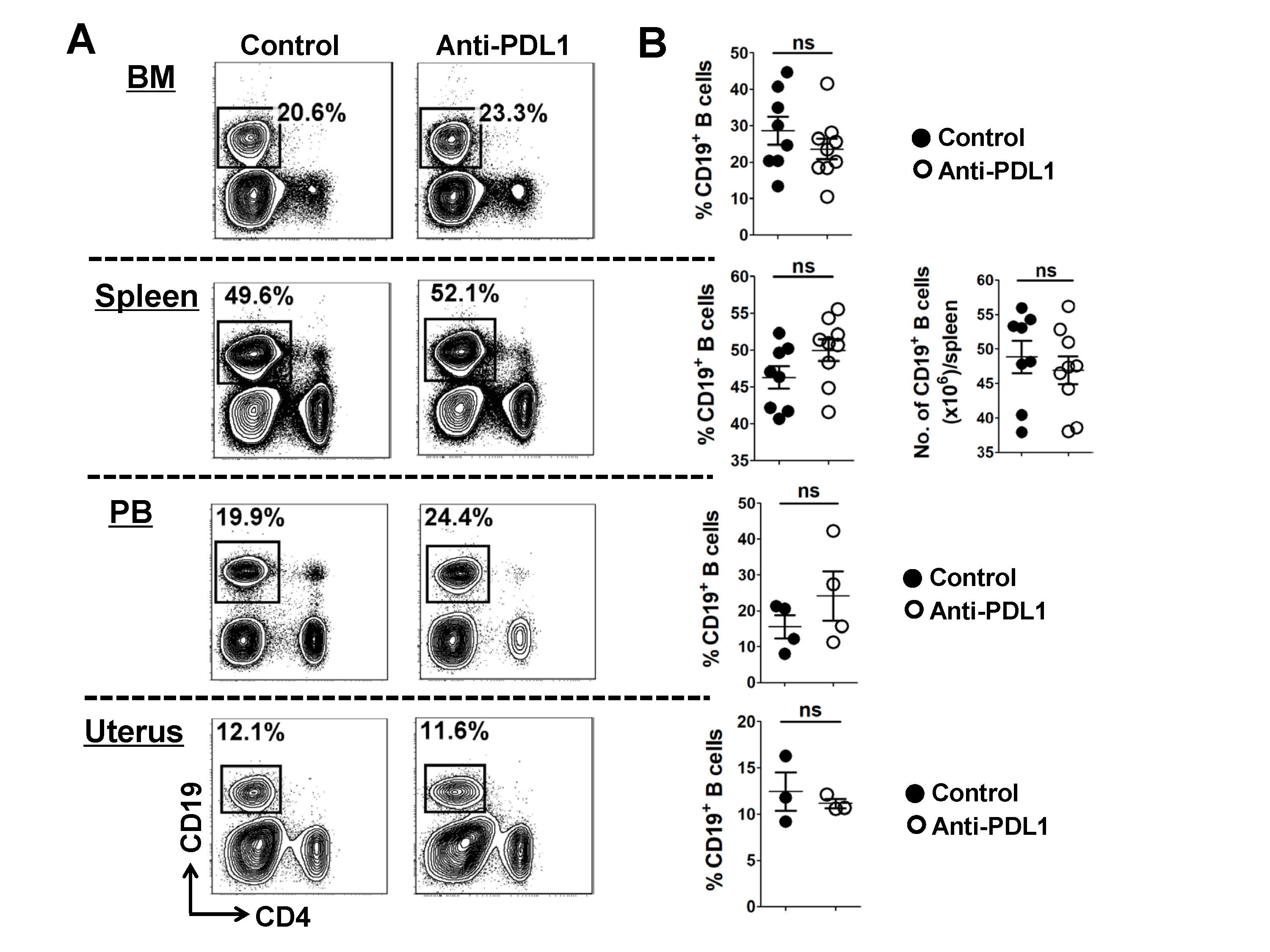

Supplement: Supplementary file 5 — Supplemental Figure 5 [file 41419_2020_2313_MOESM5_ESM.tif]

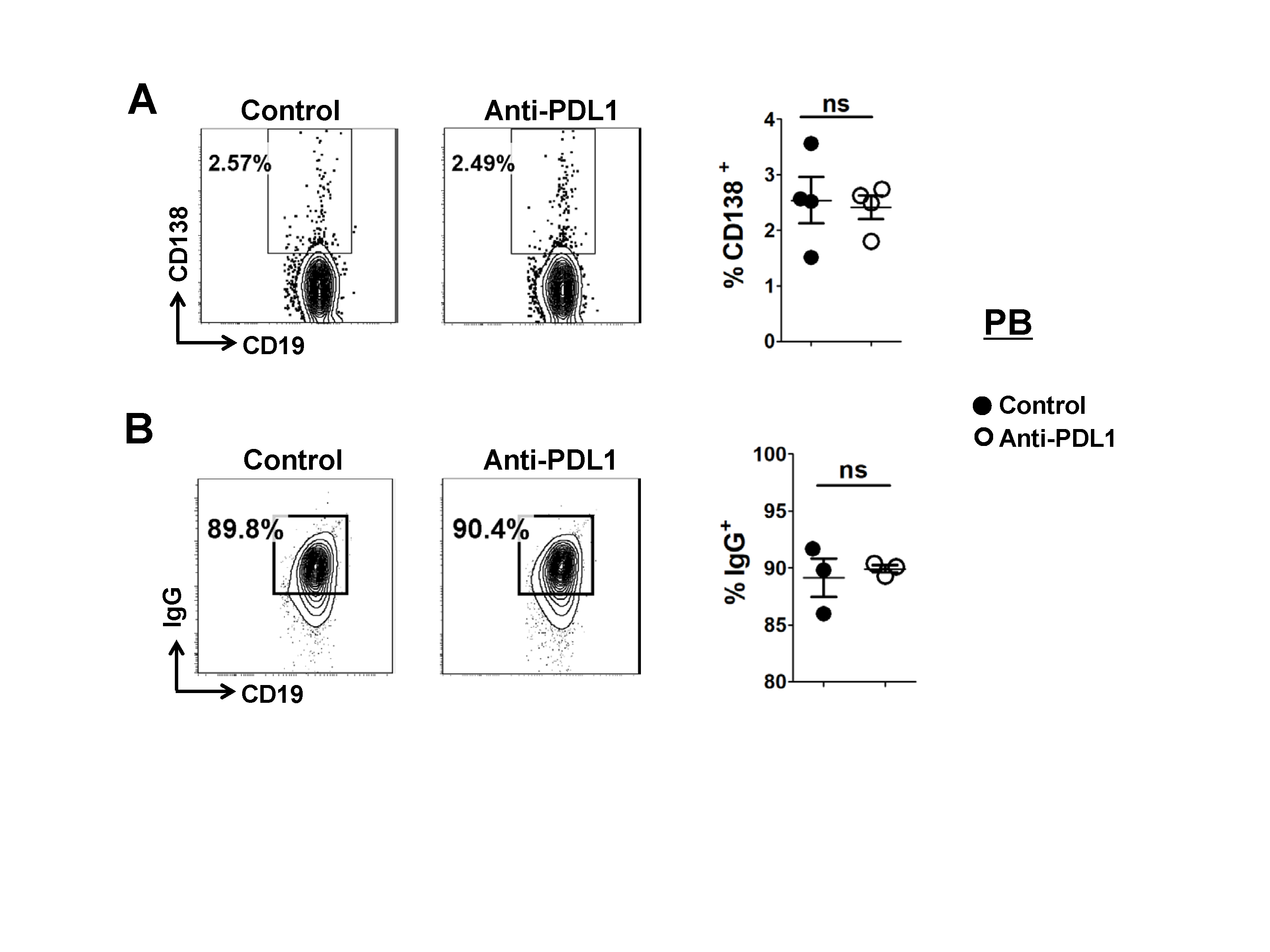

Supplement: Supplementary file 6 — Supplemental Figure 6 [file 41419_2020_2313_MOESM6_ESM.tif]

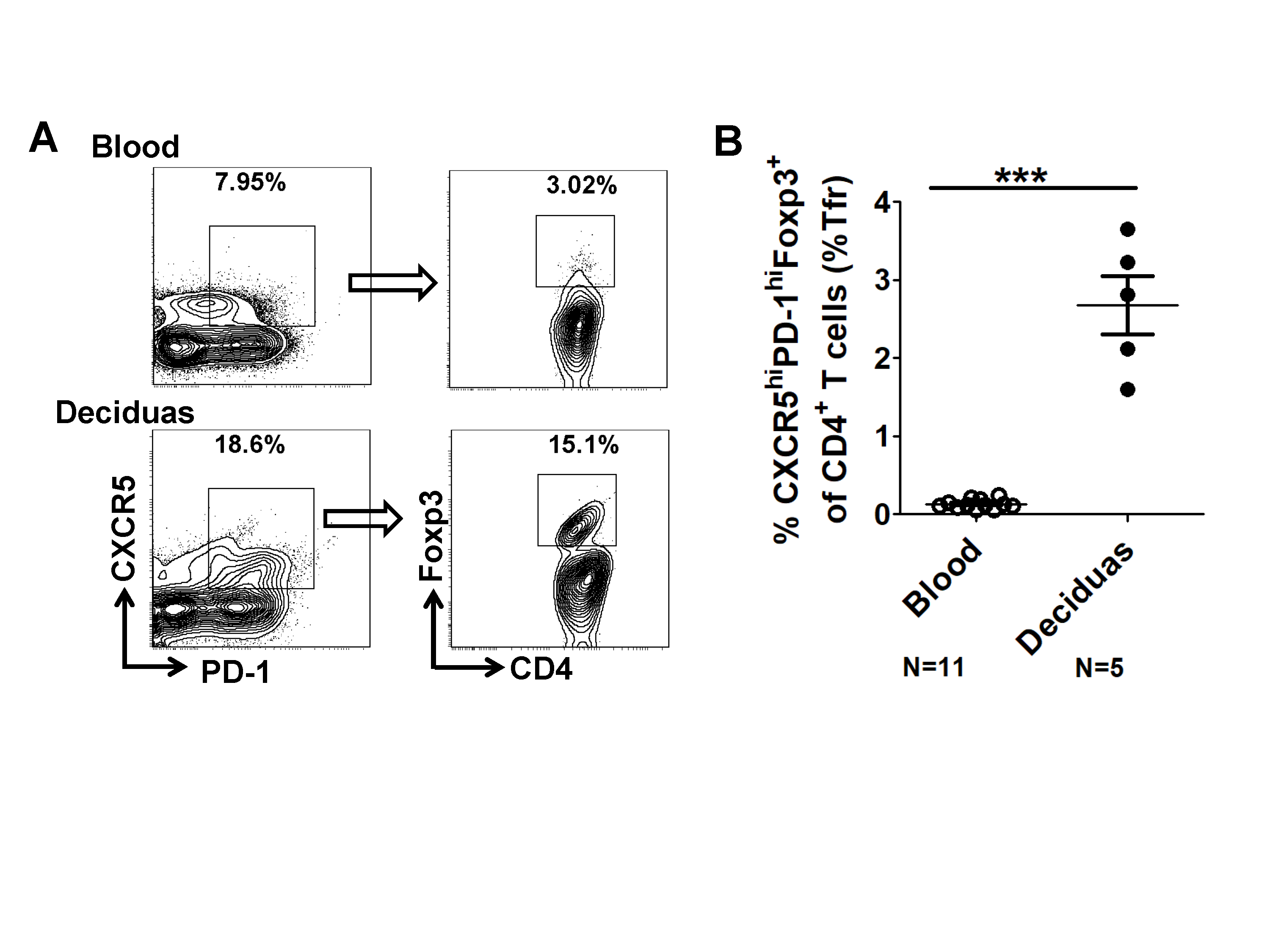

Supplement: Supplementary file 7 — Supplemental Figure 7 [file 41419_2020_2313_MOESM7_ESM.tif]
