# Supplementary material for: PDL1 blockage increases fetal resorption and Tfr cells but does not affect Tfh/Tfr ratio and B-cell maturation during allogeneic pregnancy
Source: Cell Death Dis. 2020 Feb 12;11(2):119. doi: 10.1038/s41419-020-2313-7 (PMC7016117; doi:10.1038/s41419-020-2313-7)
Supplement: Supplementary file 8 — Supplementary Figure Legend [file 41419_2020_2313_MOESM8_ESM.docx]

**Title: PDL1 blockage increases fetal resorption and Tfr cells but does not affect Tfh/Tfr ratio and B-cell maturation during allogeneic pregnancy**

**Authors:**

Weihong Zeng^1,*^, Shi Qin^1,*^, Renjie Wang^2^, Yuchen Zhang^1^, Xiaoling Ma^1^, Fuju Tian^1^, Xiao-Rui Liu^1^, Xiaoli Qin^1^, Shujie Liao^2,#^, Liqun Sun^1,#^ and Yi Lin^1,#^

**Institution:**

^1^ Shanghai Key Laboratory of Embryo Original Diseases, the International Peace Maternity & Child Health Hospital, Shanghai Jiao Tong University School of Medicine, Shanghai 200030, P. R. China.

^2^ Tongji Hospital, Tongji Medical College, Huazhong University of Science and Technology, Wuhan, Hubei 430030, P.R. China.

^*^ These authors contributed equally to the study.

^#^ **Corresponding authors:**

**Yi Lin**, Shanghai Key Laboratory of Embryo Original Diseases, the International Peace Maternity & Child Health Hospital, Shanghai Jiao Tong University School of Medicine, No. 910, Hengshan Road, Shanghai 200030, P. R. China. Telephone: +86-21-64070434. Fax: +86-21-64073421. E-mail: [yilinonline@126.com](mailto:yilinonline@126.com).

**Liqun Sun**, the International Peace Maternity & Child Health Hospital, Shanghai Jiao Tong University School of Medicine, No. 910, Hengshan Road, Shanghai 200030, P. R. China. E-mail: slq.cn@163.com.

**Shujie Liao**, Tongji Hospital, Tongji Medical College, Huazhong University of Science and Technology, Wuhan, Hubei 430030, P.R. China. E-mail: sjliao@tjh.tjmu.edu.cn.

**Running title:** Tfr cells and PDL1 blockade during pregnancy

**Supplementary Figure Legends**

**Figure S1. The uterine CD4^+^CXCR5^hi^PD-1^hi^Foxp3^+^ Tfr cells were dramatically increased at mid-gestation (E11.5) as compared with that at early-gestation (E5.5-7.5).** Adult BALB/c females were mated with C57BL/6 males, and the absolute number of CD4^+^CXCR5^hi^PD-1^hi^Foxp3^+^ Tfr cells in the uterus was determined by flow cytometry on E5.5, E7.5 and E11.5. Each symbol reflects the data from a single mouse. The data were assessed statistically using Kruskal-Wallis test. #: number; hi: high; **p < 0.01.

**Figure S2. The absolute number but not percentage of CD4^+^CXCR5^hi^PD-1^hi^Foxp3^+^ Tfr cells in the spleen is increased at mid-gestation. (A-B)** Comparison of the percentage and absolute number of CD4^+^CXCR5^hi^PD-1^hi^Foxp3^+^ Tfr cells in the spleen from NP and pregnant mice on E11.5 and E18.5. Each symbol reflects the data from a single mouse (n ≥ 13 mice per group). The data were assessed statistically using one-way ANOVA followed by Tukey’s multiple-comparison test. NP: non-pregnant; #: number; hi: high; **p < 0.01; ***p < 0.001; ns: not significant.

**Figure S3: Characterization of molecular expression in the uterine Tfr cells. (A-B)** Comparisons of intracellular BCL-6 and Foxp3 expression in Tfr cells derived from the PB, spleen, thymus and uteurs of maternal mice on E11.5. Each symbol reflects the data from a single mouse (n ≥ 6 mice per group). Geometric MFI values were calculated using FlowJo 7.6.1 software and the data were assessed statistically using Wilcoxon matched pairs test. PB: peripheral blood; MFI: mean fluorescent intensity; ns: not significant.

**Figure S4: PDL1 blockage does not affect the expression of CXCR5, Foxp3 and BCL-6 on/in Tfr cells in the spleen, blood and thymus.** **(A-C)** Comparison of the expression level of CXCR5, Foxp3 and BCL-6 on Tfr cells derived from the spleen **(A)**, blood **(B)** and thymus **(C)** of pregnant mice on E11.5 of the control and PDL1-blocked mice. Each symbol reflects the data from a single mouse (n ≥ 8 mice per group). Geometric MFI values were calculated using FlowJo 7.6.1 software and the data were assessed statistically using unpaired Student’s t-test. MFI: mean fluorescent intensity; ns: not significant.

**Figure** **S5: PDL1 blockage does not affect the proportion of CD19^+^ total B cells. (A-B)** Representative flow cytometric plots **(A)** and cumulative data **(B)** illustrating the proportion of CD19^+^ total B cells in the BM, spleen**,** PB and uterus, together with the absolute number of CD19^+^ B cells in spleen, of the control and PDL1-blocked mice. Cells are gated in lymphocytes. Each symbol reflects the data from a single mouse (n ≥ 3 mice per group). The data were assessed statistically using Mann-Whitney U test. No.: number; BM: bone marrow; PB: peripheral blood; ns: not significant.

**Figure** **S6: PDL1 blockage does not affect the** **proportion of CD138^+^ plasma cells and IgG^+^ antibody-producing B cells in the PB. (A, B)** Representative flow cytometric plots and cumulative data illustrating the proportion of CD138^+^ plasma cells (**A**) and IgG^+^ antibody-producing B cells (**B**) in the PB of the control and PDL1-blocked mice. Cells are gated in CD19^+^ B cells. Each symbol reflects the data from a single mouse (n ≥ 3 mice per group) and the data were assessed statistically using Mann-Whitney U test. PB: peripheral blood; ns: not significant.

**Figure** **S7: The proportion of CD4^+^CXCR5^hi^PD-1^hi^Foxp3^+^ Tfr cells was significantly higher in human deciduas than in peripheral blood.** Human decidual tissues (n = 5) and peripheral blood samples (n = 11) were collected from healthy women who were undergoing early elective abortions in the first trimester of pregnancy (at 6-12 weeks of gestation) at the Department of Obstetrics and Gynecology in our hospital. The proportion of CD4^+^CXCR5^hi^PD-1^hi^Foxp3^+^ Tfr cells in the deciduas and peripheral blood was determined by flow cytometry. The cells are gated in CD4^+^ T cells and each symbol reflects a sample. Data were assessed statistically using unpaired Student’s t-test. hi: high; ***p < 0.001.
