# Supplementary material for: PDL1 blockage increases fetal resorption and Tfr cells but does not affect Tfh/Tfr ratio and B-cell maturation during allogeneic pregnancy
Source: Cell Death Dis. 2020 Feb 12;11(2):119. doi: 10.1038/s41419-020-2313-7 (PMC7016117; doi:10.1038/s41419-020-2313-7)
Supplement: Supplementary file 9 — Supplemental Material and Method [file 41419_2020_2313_MOESM9_ESM.docx]

**Title: PDL1 blockage increases fetal resorption and Tfr cells but does not affect Tfh/Tfr ratio and B-cell maturation during allogeneic pregnancy**

**Authors:**

Weihong Zeng^1,*^, Shi Qin^1,*^, Renjie Wang^2^, Yuchen Zhang^1^, Xiaoling Ma^1^, Fuju Tian^1^, Xiao-Rui Liu^1^, Xiaoli Qin^1^, Shujie Liao^2,#^, Liqun Sun^1,#^ and Yi Lin^1,#^

**Institution:**

^1^ Shanghai Key Laboratory of Embryo Original Diseases, the International Peace Maternity & Child Health Hospital, Shanghai Jiao Tong University School of Medicine, Shanghai 200030, P. R. China.

^2^ Tongji Hospital, Tongji Medical College, Huazhong University of Science and Technology, Wuhan, Hubei 430030, P.R. China.

^*^ These authors contributed equally to the study.

^#^ **Corresponding authors:**

**Yi Lin**, Shanghai Key Laboratory of Embryo Original Diseases, the International Peace Maternity & Child Health Hospital, Shanghai Jiao Tong University School of Medicine, No. 910, Hengshan Road, Shanghai 200030, P. R. China. Telephone: +86-21-64070434. Fax: +86-21-64073421. E-mail: [yilinonline@126.com](mailto:yilinonline@126.com).

**Liqun Sun**, the International Peace Maternity & Child Health Hospital, Shanghai Jiao Tong University School of Medicine, No. 910, Hengshan Road, Shanghai 200030, P. R. China. E-mail: slq.cn@163.com.

**Shujie Liao**, Tongji Hospital, Tongji Medical College, Huazhong University of Science and Technology, Wuhan, Hubei 430030, P.R. China. E-mail: sjliao@tjh.tjmu.edu.cn.

**Running title:** Tfr cells and PDL1 blockade during pregnancy

**Supplemental Material and Method**

**1. Human subjects and study approval**

Human decidual tissues (n = 5) and peripheral blood samples (n = 11) were collected from healthy women who were undergoing early elective abortions in the first trimester of pregnancy (at 6-12 weeks of gestation) at the Department of Obstetrics and Gynecology in the International Peace Maternity and Child Health Hospital of China Welfare Institute (Shanghai, China). All women had never undergone preterm labor, spontaneous abortion nor preeclampsia in any pregnancy. The study was approved by the Medical Ethics Committee of the International Peace Maternity and Child Health Hospital of China Welfare Institute and all experiments were performed according to the principles of the Declaration of Helsinki. Informed consent was assigned individually from all participants before enrollment.

**2. Detailed protocols for the FCM assay**

**Part I: Intranuclear staining of Foxp3 and BCL-6**

1. Prepare cell suspensions containing approximately 1 x 10^6 cells per milliliter (ml) in flow cytometry stain buffer (FACS buffer).

Note: FACS buffer is PBS containing 3% (v/v) fetal bovine serum (FBS).

1. Divide cell suspensions into the assay tube (1.5-ml Eppendorf tube) with 1 ml per tube.
2. Spin at 1500 rpm for 5 min at 4°C, and then discard supernatant.
3. Add 100 µl of FACS buffer containing 1 µl of each fluorescein-conjugated mAb.

Note: Anti-mouse mAbs including anti-CD4-Pacific Blue, anti-CD8-V500, anti-CXCR5-PE-Cy7 and anti-CD279 (PD-1)-PE. Anti-human mAbs including anti-CD4-FITC, anti-CXCR5-PerCP/Cyanine5.5 and anti-CD279 (PD-1)-PE-Cy7.

1. Gently vortex the cells and incubate for 30 min at RT in the dark.
2. Add 1.5 ml FACS buffer to each tube, spin at 1500 rpm for 5 min at 4°C and then discard supernatant.
3. Dilute the 4 x Fixation/Permeabilization Concentrate (cat. 00-5123, eBioscience) using the Fixation/Permeabilization Diluent (cat. 00-5223, eBioscience) to the necessary volume of 1 x Fixation/Permeabilization working solution (Concentrate : Diluent = 1 : 3).
4. Add 300 µl of freshly prepared 1 x Fixation/Permeabilization working solution to each tube and vortex the cells gently.
5. Incubate the samples at 4°C for one hour in the dark.
6. Dilute the 10 x Permeabilization Buffer (cat. 00-8333, eBioscience) using distilled water.
7. Add 1 ml 1 x Permeabilization Buffer to each tube, spin at 1500 rpm for 5 min at 4°C and then discard supernatant.
8. Repeat step 11.
9. Add 100 µl of 1 x Permeabilization Buffer containing 1 µl of each intranuclear mAbs.

Note: Anti-mouse mAbs including anti-FOXP3-PE-Cyanine 5 and anti-BCL-6-APC. Anti-human mAbs including anti-FOXP3-PE and anti-BCL-6-APC.

1. Gently vortex the cells and incubate for 40 min at RT in the dark.
2. Add 1 ml PBS to each tube, spin at 1500 rpm for 5 min at 4°C and then discard supernatant.
3. Add 100 µl PBS to each tube, and transfer cells to FACS minitubes (cat. 352235, BD falcon).
4. Use BD FACS Canto II or Celestaflow cytometer (BD Biosciences, USA) to collect the immunostained cells and the FlowJo 7.6.1 software to analyze the data.

**Part II: Surface staining of B cell markers**

1. Prepare cell suspensions containing approximately 1 x 10^6 cells per ml in FACS buffer.

Note: FACS buffer is PBS containing 3% (v/v) fetal bovine serum (FBS).

1. Divide cell suspensions into 1.5-ml Eppendorf tube with 0.5 ml per tube.
2. Spin at 1500 rpm for 5 min at 4° C, and then discard supernatant.
3. Add 100 µl of FACS buffer containing 1 µl of each fluorescein-conjugated anti-mouse mAb including anti-CD19-PE, anti-GL7-Alexa Fluor 488, anti-CD95 (FAS)-PE-Cy7, anti-CD138-APC and anti-IgG (minimal x-reactivity)-PerCP-Cy5.5.
4. Gently vortex the cells and incubate for 30 min at RT in the dark.
5. Add 1.5 ml FACS buffer to each tube, spin at 1500 rpm for 5 min at 4°C and then discard supernatant.
6. Add 100 µl PBS to each tube, and transfer cells to FACS minitubes (cat. 352235, BD falcon).
7. Use BD FACS Canto II or Celesta flow cytometer (BD Biosciences, USA) to collect the immunostained cells and the FlowJo 7.6.1 software to analyze the data.

**Fluorescein-conjugated mAb Table**

| **Fluorescein-conjugated mAb** | **Source** | **Identifier** |
| --- | --- | --- |
| Anti-human |  |  |
| FITC anti-human CD4 | Biolegend | Cat#300506;RRID:AB_314074 |
| PE/Cy7 anti-human CD279 (PD-1) | Biolegend | Cat#329918; RRID: AB_2159324 |
| PerCP/Cyanine5.5 anti-human CD185 (CXCR5) | Biolegend | Cat#356910; RRID: AB_2561819 |
| APC anti-human/mouse Bcl-6 | Biolegend | Cat#358506; RRID: AB_2562472 |
| PE anti-human FOXP3 | Biolegend | Cat#320208; RRID: AB_492982 |
| Anti-mouse |  |  |
| Pacific Blue anti-mouse CD4 | BD Pharmingen | Cat#558107; RRID:AB_397030 |
| V500 anti-mouse CD8 | BD Pharmingen | Cat#560776; RRID:AB_1937317 |
| PE/Cy7 anti-mouse CD185 (CXCR5) | BD Pharmingen | Cat#560617; RRID:AB_1727521 |
| PE/Cy7 anti-mouse CD95 (FAS) | BD Pharmingen | Cat#557653; RRID:AB_396768 |
| APC anti-mouse CD138 | BD Pharmingen | Cat#558626; RRID:AB_1645216 |
| PE-Cyanine 5 anti-mouse FOXP3 | eBioscience | Cat#15-5773-80;RRID:AB_468805 |
| Alexa Fluor 488 anti-mouse GL7 | eBioscience | Cat#53-5902-82;RRID:AB_2016717 |
| PE anti-mouse CD19 | Biolegend | Cat#115508; RRID: AB_313643 |
| PE anti-mouse CD279 (PD-1) | Biolegend | Cat#109104; RRID: AB_313421 |
| APC anti-human/mouse Bcl-6 | Biolegend | Cat#358506; RRID: AB_2562472 |
| PerCP/Cy5.5 Goat anti-mouse IgG (minimal x-reactivity) | Biolegend | Cat#405314; RRID: AB_10662053 |
